# Response to ‘Feasibility of tailored treatment based on risk stratification in patients with early rheumatoid arthritis’ – authors’ reply

**DOI:** 10.1186/s13075-015-0681-7

**Published:** 2015-07-02

**Authors:** Iris M. Markusse, Willem F. Lems, Tom W.J. Huizinga, Cornelia F. Allaart

**Affiliations:** Department of Rheumatology, Leiden University Medical Center, C-01-R, PO BOX 9600, 2300 RC Leiden, The Netherlands; Department of Rheumatology, VU Medical Center, PO Box 7057, 1007 MB Amsterdam, The Netherlands; Department of Rheumatology, Reade, location dr. Jan van Breemenstraat, PO Box 58271, 1040 HG Amsterdam, The Netherlands

We thank our colleagues for their response, confirming that tailored treatment in rheumatoid arthritis patients is an important topic to address [1]. Current guidelines promote the use of prognostic factors in treatment decisions [2], but perspectives on the predicted outcome vary. Although current treatment strategies suppress radiographic progression in most patients, rapid clinical improvement (RCI) remains relevant for functional restoration [3]. We aimed to predict RCI in patients with a poor prognosis (PP) and with a nonpoor prognosis (non-PP). We based classification on prognostic factors mentioned in the European League Against Rheumatism recommendations (method 1) [2] and, for confirmation, on the Visser model (method 2), despite its focus on predicting rapid radiographic progression (RRP) [4]. We did not aim to compare both methods for superiority, but as our colleagues propose this approach we need to reiterate some of the numbers to accomplish accurate interpretation of our results.

First, of the PP patients following Visser’s model (defined as having >50 % RRP risk) receiving initial monotherapy (iMono), 46 % (not 64 %, as our colleagues mention) in fact developed RRP (Table S1 in [5]). As concluded previously, this model predicts RRP better than method 1, in which 26 % of PP patients developed RRP. However, we were interested in predicting RCI.

Second, the separation in the Health Assessment Questionnaire for PP patients versus non-PP patients treated with initial combination therapy (iCombo) (Figure S1 in [5]) is 0.11 to 0.13 per time point; that is, not clinically relevant differences [6]. However, relevant differences in the Health Assessment Questionnaire were shown after 3 months, when comparing iMono with iCombo both in PP and in non-PP patients using methods 1 and 2 (Table S1 in [5]).

Last, our colleagues attempt to compare both methods for predicting American College of Rheumatology 20/50/70 response on iCombo or on iMono in PP patients and non-PP patients. However, all odds ratios listed were determined with Visser’s method. Odds ratios determined using method 1 (Table 1) were not published previously. Comparing methods 1 and 2, the 95 % confidence intervals are largely overlapping, making it impossible to conclude that one method is superior in predicting American College of Rheumatology response without advanced statistics. These results indicate that, regardless of the method, both PP patients and non-PP patients benefit from iCombo, achieving RCI (Table 1).

**Table 1:** Odds ratios for achieving ACR response after 3 months for patients treated with initial combination therapy compared with initial monotherapy

	Method 1		Method 2 (Visser model)	
	Odds ratio	95 % confidence interval	Odds ratio	95 % confidence interval
Poor prognosis patients				
ACR20	3.94	2.09 to 7.43	10.00	3.41 to 29.32
ACR50	6.29	3.00 to 13.20	9.74	3.22 to 29.49
ACR70	7.08	2.31 to 21.70	9.33	1.97 to 44.21
Nonpoor prognosis patients				
ACR20	3.12	1.73 to 5.63	2.72	1.67 to 4.45
ACR50	6.25	3.08 to 12.70	5.39	2.98 to 9.74
ACR70	6.39	1.84 to 22.23	4.99	1.85 to 13.46

*ACR*, American College of Rheumatology

We maintain that with currently known predictors it is still impossible to define a subgroup that will achieve equal RCI on iMono as that on iCombo. Future research may reveal prognostic factors enabling us to stratify patients for differential treatment. In the meantime, more patients achieve RCI on iCombo than on iMono. This may constitute overtreatment if aiming to prevent future radiographic damage, but prevents undertreatment of debilitating arthritis already present.

## Abbreviations

iCombo: initial combination therapy
iMono: initial monotherapy
non-PP: Nonpoor prognosis
PP: Poor prognosis
RCI: Rapid clinical improvement
RRP: Rapid radiographic progression
